# Wildland fire, air pollution and cardiovascular health: is it time to focus on the microvasculature as a risk assessment tool?

**DOI:** 10.3389/fphys.2023.1225195

**Published:** 2023-07-19

**Authors:** Nazgol Naserinejad, Christy Costanian, Olivier Birot, Toussaint Barboni, Emilie Roudier

**Affiliations:** ^1^ School of Global Health, Faculty of Health, York University, Toronto, ON, Canada; ^2^ Department of Family and Community Medicine, St. Michael’s Hospital, Toronto, ON, Canada; ^3^ Muscle Health Research Center, School of Kinesiology and Health Science, Faculty of Health, York University, Toronto, ON, Canada; ^4^ Laboratoire des Sciences Pour l’Environnement (SPE), UMR-CNRS 6134, University of Corsica Pasquale Paoli, Campus Grimaldi, Corte, France

**Keywords:** wildland fire smoke, cardiovascular health, microvascular endothelial cells, microvascular geometry, particulate matter, endothelial function, population-health, airborne pollution

## Abstract

Climate change favors weather conditions conducive to wildland fires. The intensity and frequency of forest fires are increasing, and fire seasons are lengthening. Exposure of human populations to smoke emitted by these fires increases, thereby contributing to airborne pollution through the emission of gas and particulate matter (PM). The adverse health outcomes associated with wildland fire exposure represent an important burden on the economies and health systems of societies. Even though cardiovascular diseases (CVDs) are the main of cause of the global burden of diseases attributable to PM exposure, it remains difficult to show reliable associations between exposure to wildland fire smoke and cardiovascular disease risk in population-based studies. Optimal health requires a resilient and adaptable network of small blood vessels, namely, the microvasculature. Often alterations of this microvasculature precede the occurrence of adverse health outcomes, including CVD. Biomarkers of microvascular health could then represent possible markers for the early detection of poor cardiovascular outcomes. This review aims to synthesize the current literature to gauge whether assessing the microvasculature can better estimate the cardiovascular impact of wildland fires.

## 1 Introduction

Wildland fires are a major threat to human life. They adversely impact the environment, our health, and our economies. Worldwide estimates predict that global warming will generate more fire-prone areas in boreal and temperate regions. Wildland fires in these areas will become more frequent, larger, and more severe, leading to more damage and destruction (Bowman et al, 2020; Touma et al, 2021; Senande-Rivera, Insua-Costa, and Miguez-Macho, 2022). In recent years, some of the worst wildland fire seasons were reported all across the globe, with massive fires raging in Australia, in North America, around the Mediterranean Basin, in the Amazon Rainforest, and in the Siberian Boreal Forest (Lewis, 2020; Mega, 2020; Witze, 2020). In North America, including Eastern Canada (Canadian Shield and Hudson Bay), the length of the wildland fire season has increased by almost 19% over the last 35 years (Jain et al., 2018). If climate change and global warming certainly play a key role in this evolution of wildland fires (frequency, size, and severity), human factors must be acknowledged too. This was well highlighted in a recent communication from the Royal Society (Santin and Doerr, 2022): Population growth across North America increases the overall surface areas where human development intersects with wildland or vegetative fuels, thereby increasing odds of wildland fire smoke exposure to inhabitants (Peterson et al., 2021). Arson and accidental ignitions are mostly involved in highly populated areas (Balch et al, 2017). These risks can also be exacerbated by drastic fire suppression policies that aimed to subtract wildland fires from our ecosystems even though wildland fires contribute to their rejuvenation cycle. This can result in an excessive accumulation of biomass fuels (North et al, 2015).

Until recently, articles retrieved using search engines from new media database (i.e., google news, CBS, CBC, CNN and Reuters) with the keywords “wildland fire” AND “health” often focus on the immediate risks associated with wildland fires, including information about fire forecasts, injuries and casualties, losses and associated financial costs, firefighting activity, wildlife impact, and remodeled landscapes (Weber, 2020; CBS13/CNN, 2020; Canadian Red Cross, 2023; Hill, 2020). A few articles reported the short-term impact on health of chemical hazards acutely released in the air during wildland fires, having mostly an emphasis on the increased vulnerability to respiratory diseases such as asthma or more recently flu and COVID-19 (McFall-Johnsen, 2020a; McFall-Johnsen, 2020b; Kozlov, 2021). The long-term consequences of wildland fire smoke exposure remain even less documented. Long-term exposure studies have in fact essentially focused on the increased risk of developing cancer in professional firefighters (Asher-Schapiro, and Sherfinski, 2021; Kozlov, 2021). In the aftermath of Canadian wildland fires of the Spring and early Summer 2023, news media started discussing more massively the short-term impact on health of chemical hazards acutely released in the air during wildland fires (Christensen, 2023; Gibbens and McKeever, 2023; Lapid, 2023; Margaux, 2023; Migliaccio, 2023). Scientific blogs have also updated their information reporting what acute and adverse effects of wildland fire exposure are (K. Miller, 2023). These news media articles address the short-term health impact of wildland fire smoke, mostly reporting the exacerbations of respiratory symptoms and increased risks to adverse cardiovascular events.

Obviously, inhaled chemical pollutants can lodge in the respiratory tract, eventually reaching the lung alveoli at the deepest (Oberdörster, 1995; Guttenberg et al, 2016). The World Health Organization refers to particulate matters (PM) as a “common proxy indicator for outdoor air pollution”. PM_2.5_ and ultrafine particulate matters (UFP) with diameters respectively inferior or equal to 2.5 and 0.1 µm represent the smallest pollutants in size. Conversely to larger PM (as PM_10_) and other chemicals that will remain in the respiratory system, PM_2.5_ and UFP are small enough to penetrate the blood stream by passing through the lung alveolo-capillary barrier (WHO’s Air Quality and Health Unit, 2022). Subsequently, they can interact with the endothelium, the cellular lining found in our blood vessels and cardiac cavities, and we can then question their impact on cardiovascular health. This question is supported by the fact that cardiovascular disease (CVD) account for about 50% of the global burden posed by PM exposure, representing nearly sixty-one millions of disability-adjusted life years (DALYs) lost in 2019 (‘GBD Compare | IHME Viz Hub’ 2021). In the context of wildland fires, PM levels increase in the immediate vicinity of fires and also up to thousands of kilometers away from the fire center (Meng et al, 2019; Larsen et al, 2020; Matz et al, 2020; Schneider et al, 2021). In Canada between 2013 and 2018, PM originated from wildland fires in the Western and Northern ends of the continent impacted population in Central and Eastern Canada, supporting the notion of a long-range transport of wildland fire related PM.

Despite these facts, only a few recent news outlets and media release have highlighted the role that chemicals and particularly PM might play in increasing cardiovascular events (e.g., heart attacks) in response to an acute exposure to wildland fire smokes (Kozlov, 2021; Gibbens and McKeever, 2023; Lapid, 2023; Margaux, 2023; Migliaccio, 2023). Short-term exposure to wildland fire smoke seems to be a precipitating cause of adverse cardiovascular events in at-risk populations (Chen et al, 2021). Nonetheless, discrepancy exists between these studies and could be attributed to many factors: the dose exposure to PM (concentration x duration), the type of burned biomass, the use of different outcome measurements (Chen et al, 2021). Interestingly, most epidemiological studies examining the relationship between PM exposure during wildland fires and CVD use outcome measurements that are often late markers of cardiovascular conditions with a long prodromal phase, for example, hospital admissions for heart failure, myocardial infarction, pulmonary embolism, stroke. These conditions often share atherosclerosis as a common underlying cause, a pathological and aging process of the vessels developing over decades. Hence, developing effective measures to protect population from smoke exposure during wildland fire seems difficult (Hano et al, 2020; Marfori et al, 2020; Xu et al, 2020; Peterson et al., 2021).

Another important observation from these studies is the potential role played by the microvasculature. Indeed, populations with microvascular dysfunction might be more vulnerable to adverse cardiovascular outcomes in response to an exposure to wildland or biomass fire smoke. Whereas larger blood vessels (arteries, veins and venules) constitute the macrovasculature, arterioles and capillaries represent the microvasculature and the vast majority of our blood vessels. The microvasculature plays key roles in maintaining our cardiovascular health (Sörensen et al, 2016; Wagenmakers et al, 2016; Augustin and Koh, 2017; Stehouwer, 2018; Alam et al, 2019). Alterations of the microvasculature often precedes more adverse cardiovascular outcomes with macrovascular and cardiac dysfunction (Anderson et al, 2011; Lee et al, 2012; Stehouwer, 2018; van Sloten et al, 2020; Loai et al, 2022). Microvascular biomarkers that would allow an early detection of possible cardiovascular risks, when adverse effects are still reversible, could therefore represent a more suitable alternative to indicators of later CVD.

This review analyses the current literature to ascertain whether the microvascular perspective could be beneficial for the early detection of alterations in cardiovascular health during exposure to wildland fire smoke. The first section of this review (see 2.0) will determine if there is any association between microvascular alterations and cardiovascular outcomes after wildland fires based on epidemiological research. The second section of this review (See 3.0) will examine whether smoke components from wildland fires or anthropologic biomass burning impact the microvasculature. Finally, the last section (See 4.0) will identify how the remaining gaps of knowledge could be addressed to ascertain the value of microvascular biomarkers for early detection of the cardiovascular risk posed by wildland fire smoke.

## 2 What evidence do population-based studies provide regarding the impact of wildland fire on the microvasculature and cardiovascular outcome?

**FIGURE 1:**
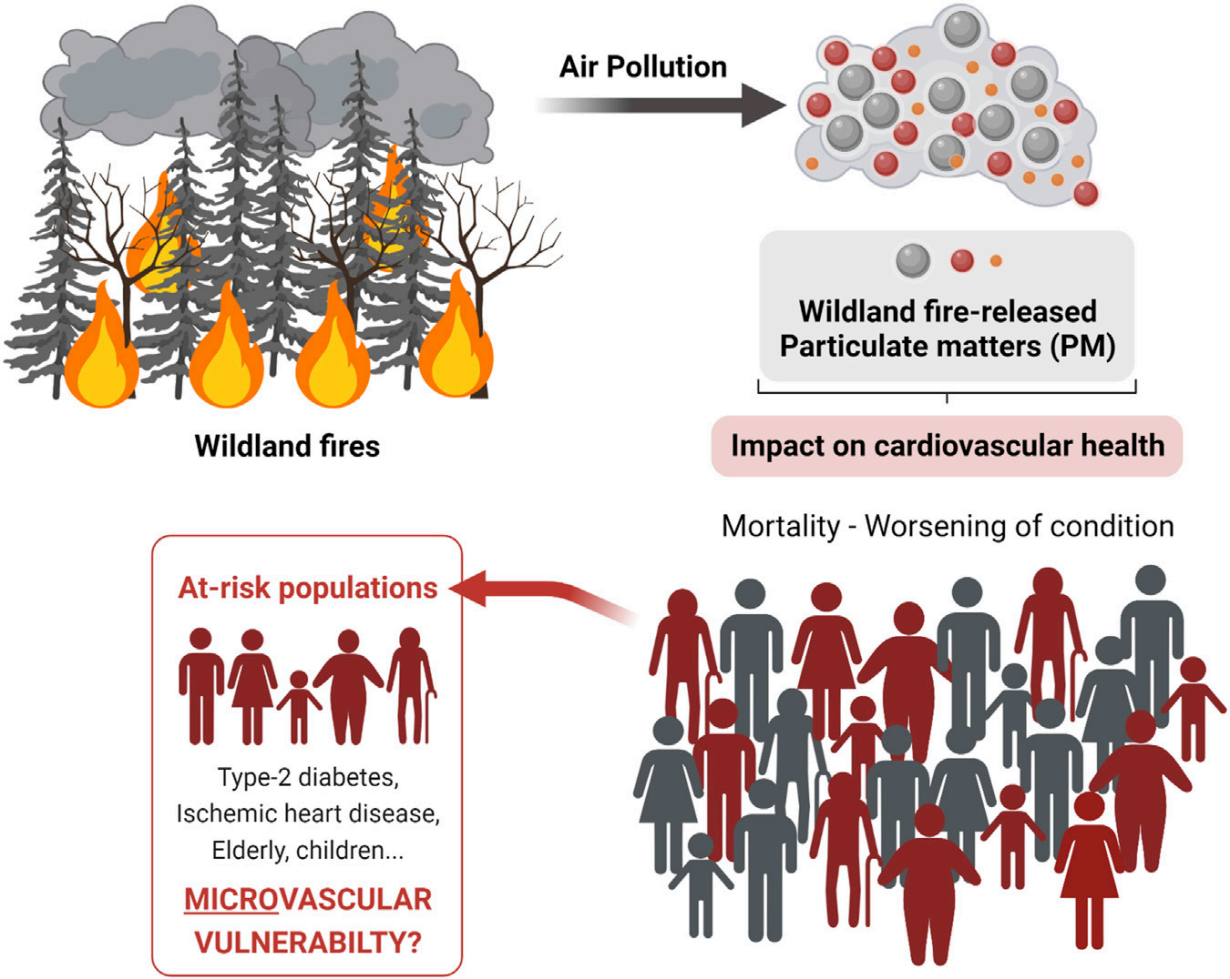
Impact of wildland fires-related PM on cardiovascular health. Population-based studies suggest that PM originating from wildland fires might more severely impact individuals with microvascular vulnerability. Created with BioRender.com.

As reviewed in (Soteriades et al, 2011; Chen et al, 2021; Reid et al, 2016; Cascio, 2018), adverse cardiovascular outcomes might be more frequent when wildland fires occur. Yet, many studies failed to demonstrate any strong relationships between PM exposure and cardiovascular risk for populations who live either far or at proximity from wildland fires (Analitis, Georgiadis, and Katsouyanni, 2012; Delfino et al, 2009; Hanigan, Johnston, and Morgan, 2008; Alman et al, 2016; Henderson et al, 2011; Liu et al, 2017; Reid et al, 2016). These equivocal results may arise from the difficulty to clearly establish the exact dose of PM a population is exposed to in the event of a wildland fire or during the entire fire season. It could also indicate that the outcomes measured are too broad to optimally assess the cardiovascular risk. Nonetheless, and noteworthy, positive association emerges more clearly in studies using cardiovascular pathologies associated with microvascular dysfunctions (e.g., diabetes or heart failure) as outcomes to test the relationship between wildland fire smoke exposure and cardiovascular risk (Figure 1). Non-invasive assessments of the microvasculature have emerged as innovative tools to stratify the cardiovascular risk at a population level, and this could be applied to wildland fires to better assess their risk for cardiovascular health.

### 2.1 Air pollution from wildland fires and biomass burning in the general population: Impact on cardiovascular mortality and medical consultations

It remains difficult to demonstrate a strong positive relationship between cardiovascular mortality and exposure to PM during wildland fire season. In the Greater Athens Area between 1998 and 2004, medium and large wildland fires (greater than $1\times 10^{6}$ m^2^ of burnt forest) were positively associated with elevated cardiovascular mortality (ICD9, 390–459) during the fire and over the following 48 h (Analitis, Georgiadis, and Katsouyanni, 2012), but this increase in mortality was not directly associated with PM_10_ exposure (Analitis, Georgiadis, and Katsouyanni, 2012). In Finland, 20 episodes of outdoor pollution due to long-range PM_2.5_ produced by biomass burning were reported in the metropolitan area of Helsinki between 2001 and 2010 (Kollanus et al, 2016). Although not statistically significant (*p* = 0.054), the outdoor concentration of PM_2.5_ was positively associated with an increased cardiovascular mortality in all ages (Kollanus et al, 2016). When analyzing the impact of wildland fires in Washington State, Doubleday and colleagues did not note significant changes in the odds of dying from cardiovascular disease on the first day of wildland fire exposure (Doubleday et al, 2020).

Analyzing the impact of wildland fires and biomass burning on medical consultations for cardiovascular events remains also largely inconclusive. When assessing wildland fire exposure based on the smoke density, medium and dense smoke significantly increased the odds of admission to emergency departments for cardiovascular and cerebrovascular diseases in Californian adults during the wildland fire season in 2015 (Wettstein et al, 2022). In Brazil, Ye and colleagues estimated that any rise of PM_2.5_ by 10 μg/m^3^ could increase admissions for cardiovascular conditions by 10% 24 h following the start of wildland fires (Ye et al, 2021). Yet, other studies did not report any positive association between PM_2.5_ and PM_10_ exposure and hospital admission due to CVD in the general population (Hanigan, Johnston, and Morgan, 2008; Kollanus et al, 2016; Mahsin, Cabaj, and Saini, 2021; Alman et al, 2016; J. C; Liu et al, 2017; Johnston et al, 2014; 2007). In Helsinki, Finland, long-range PM_2.5_ levels observed during vegetation fires were not related to any change in hospital admissions due to CVD. During the 1996–2005 fire seasons in Darwin, Australia, daily hospital admission due to CVD were not associated with PM_10_ estimates on the day of admission and up to 3 days pre-admission (Hanigan, Johnston, and Morgan, 2008). Similar observations were made in Australia in Sydney, Wollongong, and Newcastle for the 1996–2007 period, and in Western regions of North America (British Columbia and California) during Summer 2003. During fire seasons across various settings, PM_2.5_ or/and PM_10_ levels were not related to hospital admission or physician visits due to overall CVD in the general population (Delfino et al, 2009; Henderson et al, 2011; Martin et al, 2013; Johnston et al, 2014; Hutchinson et al, 2018; Doubleday et al, 2020). Yet, some of these studies reported positive association with respiratory diseases admission (Hutchinson et al, 2018; Doubleday et al, 2020).

The above studies did not identify any clear relationship between exposure to PM from wildland fires or biomass burning and medical consultations for cardiovascular pathologies or cardiovascular mortality in the general population.

### 2.2 Impact of air pollution from wildland fires and biomass burning in patients presenting microvascular vulnerability

When analyzing specific cardiovascular conditions and age groups, it appears that certain populations with microvascular dysfunction might be more vulnerable to adverse cardiovascular outcomes. Older individuals and patients with cardio-metabolic diseases, such as diabetes, heart failure, and ischemic heart diseases, were more likely to seek medical assistance following exposure to wildland or biomass fire smoke (Delfino et al, 2009; Henderson et al, 2011; Haikerwal et al, 2015; Yao et al, 2020; Mahsin, Cabaj, and Saini, 2021; Wettstein et al, 2022). It is noteworthy that these cardio-metabolic diseases are associated with microvascular alterations that can alter whole-body performance. Heart failure and ischemic heart disease present functional alterations of the heart and skeletal muscle microvasculature (D’Amario, Borovac, and Crea, 2021; Kitzman et al, 2014; D’Amario et al, 2019; Mehta et al, 2022; van de Hoef et al, 2020). Patients unable to maintain a functional microvasculature in the skeletal muscle and cardiac tissues have a greater intolerance to performing daily activities, that could worsen symptoms such as dyspnea and fatigue that are hallmarks of heart failure and main causes of hospitalizations (Kitzman et al, 2014; Del Buono et al, 2019; Mahfouz, Gouda, and Abdelhamid, 2020). As individuals age, microvascular dysfunctions in striated cardiac and skeletal muscles will limit blood flow distribution to these tissues during physical exertion, making them more susceptible to ischemic pathologies (Bearden, 2006; A. J; LeBlanc and Hoying, 2016).

Multiple studies have reported an increased risk to seek medical care due to acute myocardial infarction, ischemic heart disease, and congestive heart failure in the event of wildland fire-related PM exposure (Johnston et al, 2014; 2007; Rappold A. G. et al, 2011; Rappold et al, 2012; Haikerwal et al, 2015; Yao et al, 2020; Mahsin, Cabaj, and Saini, 2021; Wettstein et al, 2022). The risk appears to be even higher for older individuals (Delfino et al, 2009; Johnston et al, 2014). For example, during the Fall 2003 Californian wildland fire season, PM_2.5_ exposure was positively associated with hospitalization when examining admissions due to congestive heart failure (+11.3%, all age groups) for individuals aged 45–99 years (Delfino et al, 2009). In Australia, during bush fires, PM exposure led to higher odds of emergency department admissions due to ischemic heart diseases in Darwin’s aboriginal population and due to heart failure in Sydney’s inhabitants (Johnston et al, 2014; 2007). During the wildland fire season of Summer 2006–2007 in Victoria (Australia), any increase of PM_2.5_ by 9 μg/m^3^ increased the risk of hospital admission due to ischemic heart disease and acute myocardial infarction (Haikerwal et al, 2015). Similar observations were made during the fire seasons of 2010–2015 in British Columbia (Canada) where increased odds of myocardial infarction (+19% after 17 h) and ischemic heart disease (+7% after 28 h) following exposure to PM_2.5_ were reported (Yao et al, 2020). An increased risk of diabetic outcomes (i.e., hyperglycemia or hypoglycemia) (+20%) was also observed 48 h post-exposure (Yao et al, 2020). Some of the studies cited above indicate that older individuals aged 65 years and older had a higher risk for hospital admission due to CVD (Haikerwal et al, 2015; Mahsin, Cabaj, and Saini, 2021; Wettstein et al, 2022). Every increase in PM_2.5_ by 10 μg/m^3^ increased the risk of physician visits due to congestive heart failure and ischemic heart disease by 11% and 19%, respectively, for seniors aged 65 years and older. To add, seniors with diabetes had an increased risk of cardiovascular morbidity (+30%), post fire (Mahsin, Cabaj, and Saini, 2021). In California, older individuals (65+) had a greater risk of admission due to cardiovascular and cerebrovascular diseases when the density of wildfire smoke increased above 10.5 μg/m^3^ of PM_2.5_ (Wettstein et al, 2022). While the association between PM exposure and cardiovascular risk in the general population remains uncertain, exposure to wildland fire smoke appears to be linked with worse cardiovascular outcomes in older individuals and in patients with other comorbid cardiometabolic diseases (i.e., heart failure, ischemic heart diseases and diabetes). Since microvascular dysfunction worsens cardiometabolic symptoms, it may be postulated that exposure to wildland fire smoke may directly trigger additional microvascular dysfunction thereby exacerbating pre-existing cardiovascular morbidities.

### 2.3 Direct evidence of the impact of wildland fires or biomass burning exposure on the microvasculature in the general population

A limited number of studies have investigated whether outdoor air pollution generated by wildland fires could directly impact the microvasculature. Yet, a few studies have non-invasively evaluated the microvasculature, corroborating the notion that biomass burning could indeed impact the microvasculature, both functionally and structurally. *In vivo* imaging of the retina allows for a non-invasive assessment of microvascular alterations. Changes in the geometry of the retinal microvasculature has indeed emerged as a valuable tool to stratify cardiovascular risk. A reduced diameter of the central retinal arterioles (CRAE), a greater diameter of the central retinal venules (CRVE) and a lower arteriolar-to-venular ratio (AVR = CRAE/CRVE) are associated with adverse cardiovascular events and cardiovascular risk factors (Mutlu et al, 2016; Seidelmann et al, 2016; Rijks et al, 2018). Retinal microvasculature measurement has been used to assess the impact of urban airborne pollution on microvascular function among individuals. This approach has shown that long-term and short-term exposure to black carbon, PM_2.5_ and PM_10,_ was linked with a decreased central retinal arteriole diameter among adults and children (Adar et al, 2010; Louwies et al, 2013; Provost et al, 2017). Thus, urban air pollution alters the geometry of the retinal microvasculature, potentially suggesting an increased cardiovascular risk. Among children aged 4–6 years, prenatal and early childhood exposure to PM_2.5_ and nitrogen oxide (NO_x_) was associated with widening of the retinal venular and arterial diameters (Luyten et al, 2020). Using a similar approach, Korsiak and colleagues assessed the short-term impact (same day to 3 weeks of lag exposure) of outdoor pollution from biomass burning on the retinal microvasculature in children aged 4–12 years living in rural areas (Korsiak et al, 2021). PM_2.5_ levels and an index of the presence of oxidant gases (Ox) that combines a measure of ozone and nitrogen oxide (O_3_ and NO_x_) to assess outdoor pollution. Any increase of Ox by 10 ppm over a period of 7 days was associated with a decrease in retinal arteriole diameter by 2.63 μm (−1.44% of the total arteriolar diameter). Yet, PM_2.5_ exposure due to biomass burning only impacted the diameter of retinal arterioles when Ox levels were high (Korsiak et al, 2021), suggesting that the interaction between oxidant gases and PM elicits greater impact on the microvasculature. Further investigations will better delineate whether structural changes observed in children’s retinal microvasculature are good predictors of future cardiovascular risks.

Another approach has evaluated whether residential pollution alters cutaneous microvascular functions. Measuring changes in skin blood flow is a promising non-invasive tool to evaluate the microvascular function, and to indirectly assess cardiovascular health. In fact, Witters and colleagues assessed the impact of residential air pollution on the cutaneous microvascular function of children aged 4–6 years using local heat-mediated vasodilation (Witters et al, 2021). In response to local skin heating, vasodilation of the skin’s microvasculature increases local blood flow as measured by laser doppler. Prenatal exposure to black carbon, PM_2.5_ or PM_10_ of residential origin during the last trimester of pregnancy lowered the cutaneous microvascular function in children aged 4–6 years (Witters et al, 2021). Yet, post-natal exposure had no impact on the microvascular function and did not modify the association between prenatal exposure and microvascular function (Witters et al, 2021). To our knowledge, the impact of PM exposure on skin microvascular function has not been investigated in the specific context of wildland or biomass burning.

As conclusion of this first section, future studies combining an accurate dosimetry of PM with a non-invasive assessment of the microvascular function would contribute to better evaluate the cardiovascular risk of wildland fires PM exposure.

## 3 Could chemicals present in wildland fire smoke impact the microvascular endothelium?

### 3.1 Overview of the microvasculature

The vasculature is a vast system of blood vessels composed of arteries, arterioles, capillaries, venules, and veins. All these vessels are primarily composed of a layer of endothelial cells forming the endothelium. Arterioles and capillaries represent together the microvasculature whereas larger diameters vessels constitute the macrovasculature. Arterioles are located downstream of resistance arteries. In a simplistic representation, arterioles can be seen as vascular tubes made of endothelial cells and supported by vascular smooth muscle cells (VSMCs) that confer to arterioles their vasomotricity (vasoconstriction and vasodilation). Such vasomotricity allows arterioles to play a central role in controlling peripheral vascular resistance and blood pressure (Jackson, 2021). For example, in the skeletal muscle, which represent about 40% of the body mass, arterioles contributes for about 50% of tissue vascular resistance (Fronek and Zweifach, 1975). These arterioles largely determines the blood flow into downstream capillaries, which are the most abundant and smallest blood vessels in the human body. Capillaries are constituted of a single layer of endothelial cells, supported by mural cells such as pericytes. In a healthy adult, the capillary endothelium with a surface of 600 m^2^ represents more than 85% of the total vascular surface (Wagenmakers et al, 2016). The capillary endothelium supports key homeostatic functions: delivery of oxygen and nutrients to tissues, capacity of tissues to respond to infection, metabolic tissular adaptation, thermoregulation, endocrine communication, tissue regeneration after injury (Augustin and Koh, 2017). The microvascular endothelium found in arterioles and capillaries also senses local vasodilatory molecules present in the tissue, relaying the information to the arteriolar VSMCs. The coordinated action of the microvascular endothelium and smooth muscle tissue regulates the process of arteriolar vasodilation ensuring an appropriate blood supply to the tissue as well as an optimal control of blood pressure (Murrant and Sarelius, 2015; Dora, 2016; Wagenmakers et al, 2016). From this standpoint, it is obvious to comprehend why health, and particularly cardiovascular health, requires an optimal microvasculature (Figure 3). Recent studies have suggested that atmospheric pollution and PM could dysregulate blood pressure. Wen and colleagues have reported a positive association between atmospheric pollution and diastolic blood pressure in post-menopausal women (Wen et al, 2023). And Marrone and colleagues have described a potential positive correlation with systolic blood pressure in adolescents (Marrone et al, 2023). Based on these observations, studying whether exposure to wildland fire smoke has a short-term or long-term impact on the microvasculature has become more critical than ever.

### 3.2 Presence of inhaled pollutants in the blood circulation and interaction with the endothelium

**FIGURE 2:**
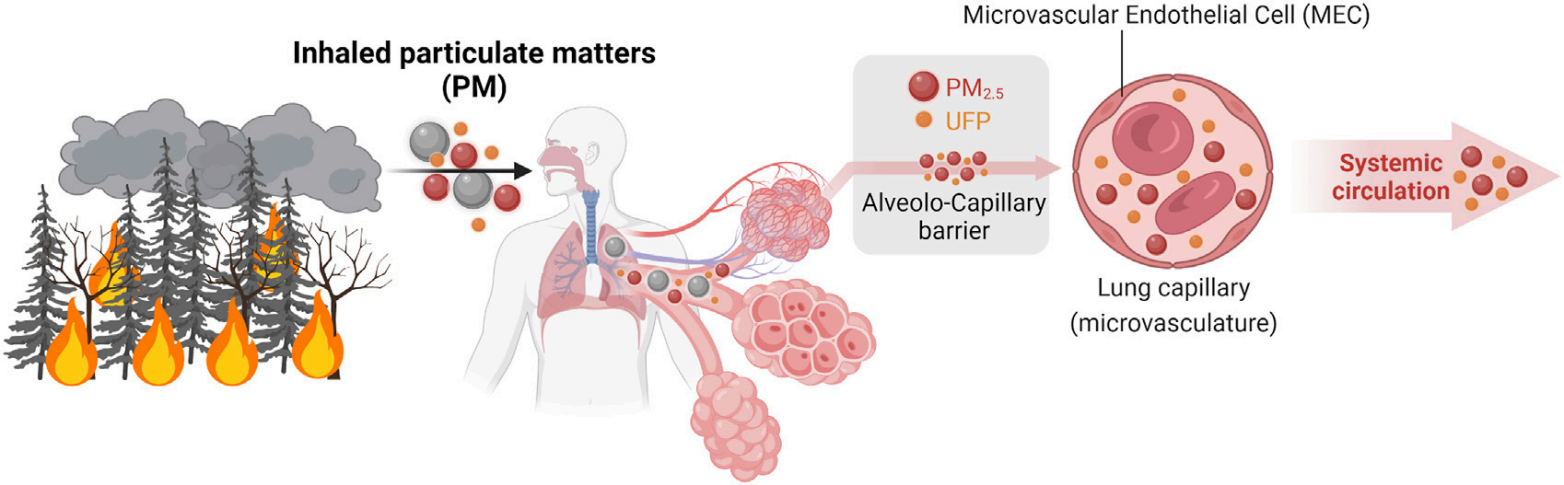
Wildland fire-related PM can enter the bloodstream. Being the smallest pollutants, PM_2.5_ and ultrafine PM (UFP) penetrate the alveolo-capillary barrier and then enter the bloodstream. Created with BioRender.com.

To impact the endothelium, and more particularly the microvascular endothelium, wildland fire pollutants must enter the blood stream (Figure 2). Biomass burning and wildland fire emit smoke into the atmosphere that contain a complex mixture of chemicals under the form of gases, volatile organic and semi-volatiles compounds (VOC and SVOC), polycyclic hydrocarbons, and particulate matter (Barboni et al, 2011; 2010; Romagnoli et al, 2014) (Baxter et al, 2014; Navarro et al, 2019; 2017; Cherry et al, 2021). The main pollutants released are carbon dioxide (CO_2_), carbon monoxide (CO), nitrogen oxides (NO_x_), and aerosols (i.e., particulate matter). Aerosols consist of solid particles, such as soot, with a diameter less than or equal to 1 µm (UFP and PM_1_), and liquid particles in the form of tar, with a diameter less than or equal to 2.5 µm (PM_2.5_). Aerosols are molecules with long-range transportability that can be inhaled during breathing and absorbed through skin deposition during or in the aftermath of a wildland fire (Meng et al, 2019; Cherry et al, 2021).

**FIGURE 3:**
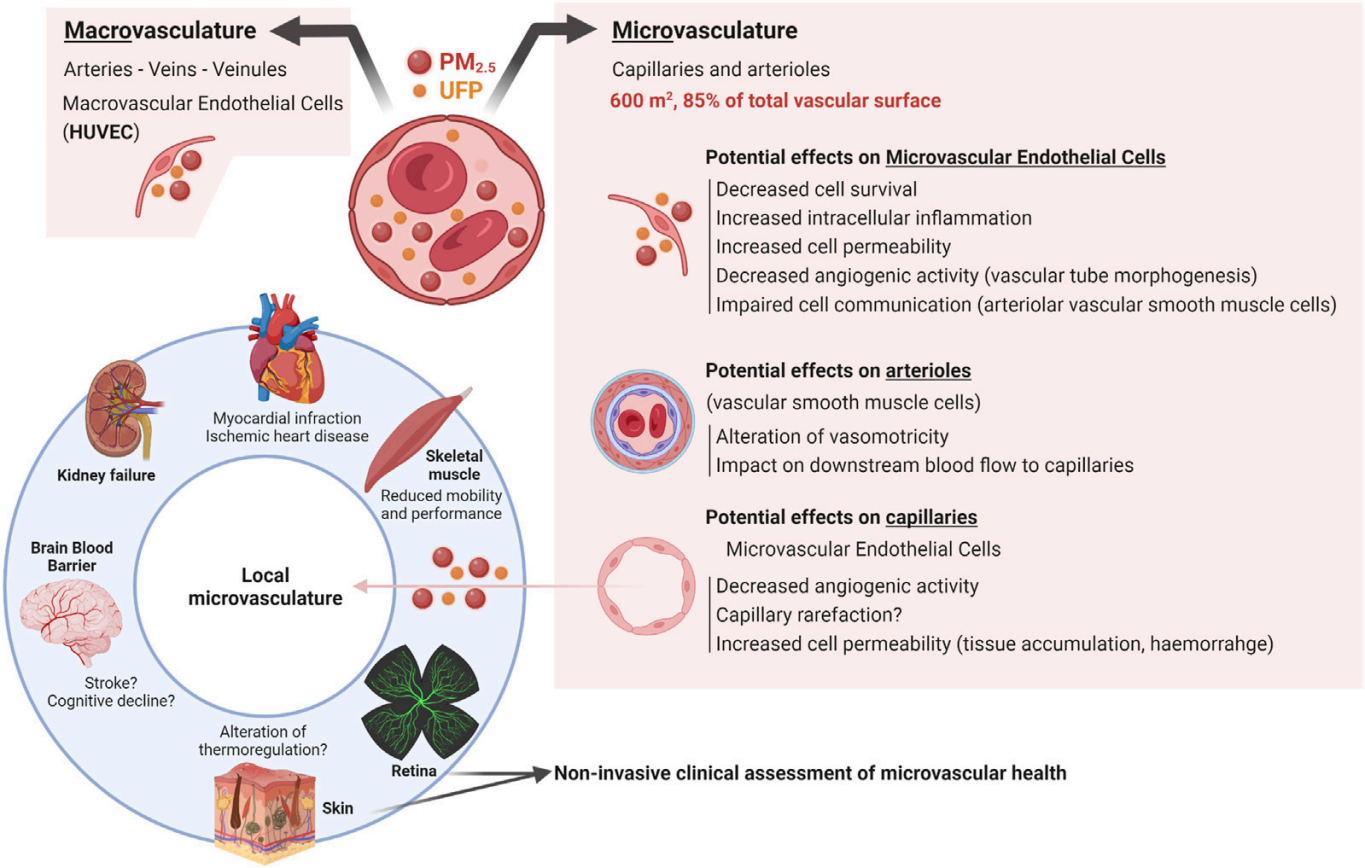
Circulating PM interacts with macro- and micro-vascular endothelia potentially impacting multiple tissues. Once in the bloodstream, PM_2.5_ and ultrafine PM (UFP) might impair the biology of macro- and micro-endothelial cells with consequences on vessel integrity (arterioles, capillaries), angiogenic capacity, and inflammation. The microvasculature of various tissues and organs (kidneys, cardiac and skeletal muscle, brain, skin, retina) could be altered with consequences on disease progression and functionality (cognitive aging, fatigue, worsening of cardiac conditions). Non-invasive assessments of microvasculature could represent a new avenue for cardiovascular risk assessment in the context of wildland fire smoke exposure. Created with BioRender.com.

As previously mentioned, the smallest PM (PM_2.5_ and UFP) can penetrate through the lung alveolo-capillary barrier into the blood stream (Figure 2). Once in the circulation, these PM promote systemic inflammation, affecting both circulating immune cells and endothelial cells (Pope et al, 2016; Finch et al, 2019). Limited studies have specifically investigated whether PM_2.5_ and UFP directly interact with the microvascular endothelium. Yet, a few studies support this notion. First, inhaled nanoparticles were found to circulate in the blood stream and accumulate in diseased arteries in both preclinical rodent models and in human subjects (Miller et al, 2017; Raftis and Miller, 2019; Nemmar et al, 2002). A few studies have also shown that after inhalation, nanoparticles circulate in the microvascular beds of multiple organs. For example, inhaled silica nanoparticles were found in capillaries from lung, lymph nodes, spleen, and kidney tissues from male F344 rats (Guttenberg et al, 2016). Additionally, an accumulation of PM_2.5_ originating from organophosphate flame retardants was observed in the heart, brain, and skeletal muscle tissues from C57B6J mice (M. Chen et al, 2020). The endothelia in these tissues are continuous. To reach tissue cells, these circulating pollutants must therefore interact with and then cross the microvascular endothelium to penetrate and accumulate into tissues. The interaction between these circulating PM and endothelial cells could also modify the communication between endothelial cells and VSMCs and alter the arteriolar vasomotricity with consequences on the blood flow feeding downstream capillaries (Figure 3). The impact of PM on the vasomotricy might vary depending on the size of PM inhaled. Using calibrated titanium oxide PM, Nurkiewicz et colleagues reported that the microvascular impairment (i.e., capacity of the arterioles to dilate) increases as the size of PM decreases (Nurkiewicz et al, 2011). Once in these tissues, PM can promote cell damages and oxidative stress that trigger inflammation (Farina et al, 2019; Hameed et al, 2022). This can feed a vicious circle where tissue inflammation could promote further microvascular dysfunction (Nurkiewicz et al, 2011; 2008). Circulating PM could also interact with the capillary endothelium to impair the capacity of the capillary bed to remodel or grow through angiogenesis, the formation of new capillaries from pre-existing ones (Abe et al, 1990; Capitão and Soares, 2016; Fuchs et al, 2017; Shi and Vanhoutte, 2017) (Figure 3). Pope and colleagues have analyzed the impact of urban-related PM and UFP on the blood composition (Pope et al, 2016). They reported that PM_2.5_ concentration correlated positively with the number of circulating endothelial microparticle, a marker of endothelial cell damage, and negatively with the concentration of circulating cytokines known to promote angiogenesis (the formation of new capillaries from existing ones). To date, it remains unknown whether PM from wildland fire origin have similar impact on the microvasculature. While it remains unclear if a similar effect (tissue accumulation) prevails with PM specifically released from wildland fire smoke, these findings suggest that ultrafine and fine PM from airborne pollution could circulate through the microvasculature, interact with microvascular endothelia, and accumulate in various tissues (Figure 3). If confirmed, these PM-induced microvascular alterations could have important repercussions on cardiovascular health since arteriolar vasomotricity and capillary angiogenesis could *de facto* support the restoration of blood flow and tissue repair post-ischemic events (e.g., myocardial infarction, peripheral arterial disease). Indeed the alteration of these microvascular processes can participate to the pathogenesis and progression of CVD (Lähteenvuo and Rosenzweig, 2012; Crea, Montone, and Rinaldi, 2021; D’Amario, Borovac, and Crea, 2021; Dean et al, 2015; Annex and Cooke, 2021; Coker et al, 2019; Chen et al, 2016).

### 3.3 Assessing the impact of wildland fire smoke on the microvascular smooth muscle

Limited literature exists regarding the impact of PM from wildland fire smoke on vascular smooth muscle cells (VSMCs). As reviewed by Ming and colleagues, the impact of PM_2.5_ on VSMCs has been mainly investigated for particles originating from cigarette smoke, diesel exhaust, or urban airborne pollution but not wildland fires and other biomass burning (Ming et al, 2022). In addition, most of these studies have used smooth muscle cells from macrovascular origin (i.e., isolated from aorta) with an interest in studying whether PM could promote atherosclerosis through the activation of smooth muscle hypertrophy (Chang et al, 2017; Wan et al., 2018; Ho et al, 2019; Gu et al, 2021; Ho et al, 2021; Tian et al, 2021; Ju et al, 2022; Ming et al, 2022; Bao et al, 2023). Similarly to cigarette smoke-related PM, particles from urban pollution increase mitogenic and hypertrophic signals (e.g., PDGFR-β, JAK2/STAT3) in macrovascular VSMCs, promoting the thickening of the media, an contributing to atherosclerosis and calcification in the aorta (Bao et al, 2023; Schroeter et al, 2008; R; Li et al, 2013). Exposure to PM from cigarette smoke and urban airborne pollution also induce an important oxidative stress in these macrovascular VSMCs, enhancing pro-inflammatory signals (i.e., NFκB), the expression of metalloproteinase (MMPs), cell proliferation and migration (Chang et al, 2017; Ho et al, 2021; Ju et al, 2022). These studies indicate that PM can significantly alter the phenotype of VSMCs. PM exposure has been reported to significantly impair the microvascular function, with frequent report of endothelial dysfunction (Pope et al, 2016; Nurkiewicz et al, 2004; A; LeBlanc et al, 2009). Yet, nanoparticles formed of titanium oxide altered the endothelial function in sub-epicardial arterioles without apparent changes in the sensitivity of the vascular smooth muscle to nitric oxide, the most potent local vasodilator (LeBlanc et al, 2009). We could therefore hypothesize that PM produced by wildland fires could have a similar impact of the phenotype of microvascular VSMCs. To our knowledge, there is no study that has investigated the impact of PM from wildland fire smoke on VSMCs from microvascular origin. It remains unknown what the impact of wildland fire related PM on the capacity of arterioles to dilate could be. This highlight the need to perform further investigation on whether wildland fire smoke could alter the control of blood flow by damaging the microvascular smooth muscle.

### 3.4 Assessing *in vitro* the impact of wildland fire smoke pollutants on microvascular endothelial cells

Despite the importance of the microvasculature in cardiovascular health, Microvascular Endothelial Cells (MECs) remain largely understudied as an *in vitro* model to investigate the effect of airborne pollutants on endothelial cells. The most frequently used endothelial cells to investigate the toxicity of nanoparticles and air pollution are macrovascular Human Umbilical Vein Endothelial Cells (HUVECs) (Ding et al, 2020; S; Chen et al, 2017; He et al, 2021; Setyawati et al, 2015; Jung et al, 2022; Su et al, 2020; Hu et al, 2017; Y; Liu et al, 2021; Wang et al, 2017), which are phenotypically different from MECs, and therefore might respond differently to stressors (Lang et al, 2003; Suphasiriroj, Mikami, and Sato, 2013; Lau et al, 2021). This might hold true for PM, as nanotoxicity is known to vary depending on the phenotype and shape of endothelial cells (Setyawati et al, 2015). When comparing the impact of urban PM_2.5_ on HUVECs and human microvascular ECs (HMECs), Chen and colleagues reported a decrease in cell survival, an increase in intracellular inflammation, and reduced angiogenic capacity for high concentrations of PM_2.5_ (100–800 μg/mL, 24 h treatment) in both microvascular (HMECs) and macrovascular (HUVECs) endothelial cells (Chen et al, 2017). Yet, the effects of PM_2.5_ were less severe in HMECs. In another study, exposure to PM_10_ (5–50 μg/mL for 24 h) altered cell permeability in human brain MECs (HBMECs) and led to the acquisition of pro-senescent and pro-inflammatory profiles with high levels of ICAM and vascular adhesion molecule-1 (VCAM) in these cells (Park et al, 2021).

In spite of the limitation of being of macrovascular and venous origin, HUVECs have nonetheless proven useful to assess chemicals toxicity (Cao et al, 2017; Cao, 2018). Nanoparticles and PM_2.5_ can enter HUVECs through endocytosis and micropinocytosis and can be released by exocytosis (Cao, 2018; Su et al, 2020). The mechanism of cell internationalization varies depending on the chemical composition of the PM_2.5_. For example, metal-rich PM_2.5_ enter HUVECs through micropinocytosis; PAH-rich PM_2.5_ require clathrin-dependent endocytosis, and water-soluble PM_2.5_ are internalized through a caveolin-dependent endocytosis (Su et al, 2020). Once inside the cells, PM_2.5_ can alter the cytoskeleton structure and reduce HUVECs survival via apoptosis in a time and dose dependent manner (Wang et al, 2017; Su et al, 2020). Beyond inducing cell apoptosis, PM_2.5_ can also alter endothelial cell functions such as cell permeability, inflammation, and angiogenesis. In a tridimensional culture model, HUVECs exposed to PM_2.5_ showed an increased expression of the pro-inflammatory markers interleukin-1 (IL-1), Nuclear Factor Kappa-light-chain-enhancer of activated B cells (NF-κB), and intercellular adhesion molecule-1 (ICAM-1) (Y. Li et al, 2019). PM_2.5_ composed of organic carbon and element carbon were shown to increase vascular permeability in HUVECs (Long et al, 2020).

PM_2.5_ exposure can also slow down the process of autophagy and increase activity of the tumor suppressor gene p53 (TP53) in HUVECs (Liu et al, 2021; Wang et al, 2017). Autophagy is an important intracellular process that identifies and degrades unnecessary or dysfunctional proteins and organelles. Autophagy has protective functions against cytotoxic actions of microenvironmental stressors promoted by metabolic risk factors (e.g., high glucose; oxidized low density protein, ox-LDL) (Jiang, 2016). An overactivation of tumor suppressor protein 53 (TP53) in the endothelium can challenge cardiovascular homeostasis, making arteries more prone to atherosclerosis (Warboys et al, 2014). Angiogenesis, the formation of new capillaries from pre-existing ones, supports the expansion of the microvascular network to restore oxygen and nutrient supply to tissues during cardiac hypertrophy and after post-ischemic events (e.g., myocardial infarction, peripheral arterial diseases) (Li, Zhao, and Zhu, 2022; Annex and Cooke, 2021; Dorn, 2007; Sano et al, 2007). Some of the identified cellular effects of PM_2.5_ could alter the angiogenic process. For example, previous reports indicate that an increase in TP53 during ischemia limits angiogenesis during cardiac hypertrophy and in preclinical model of peripheral arterial diseases (Gogiraju et al, 2015; Pfaff et al, 2018).

Two toxicogenomic studies bring further evidence that PM_2.5_ could alter the angiogenic capacity of HUVECs (Hu et al, 2017; Jung et al, 2022). After 24 h of exposure to 50 μg/mL of urban airborne PM_2.5_, 501 genes were differentially regulated, 177 genes were downregulated and 417 genes were upregulated (Hu et al, 2017). In the second study, 24 h of exposure to PM_2.5_ originated from gasoline engine exhaustion (59.0 μg/mL) changed the expression of 1081 genes associated with cardiovascular system morphogenesis, vascular tube formation, receptor signaling (cytokine-related signaling), or cell adhesion (Jung et al, 2022). Both studies suggest that PM_2.5_ exposure could in fact alter important processes for angiogenesis such as morphogenesis, vascular tube formation, receptor signaling. Yet, many of the top differentially regulated genes differs between the two studies (Hu et al, 2017; Jung et al, 2022). The observed discrepancy might result from the difference in chemical compositions of the PM_2.5_. One study used PM_2.5_ originated for gasoline exhaustion (100%) (Jung et al, 2022), while the second versus has PM_2.5_ of mixed origin from both coal combustion and vehicle exhaustion (Hu et al, 2017; Y; Liu et al, 2021). Altogether, these endothelial cell alterations induced by PM_2.5_ and other pollutant nanoparticles could make the vasculature more vulnerable to cardiovascular risk factors and therefore, prone to atherosclerosis development.

Finally, if the observations made in HUVECs were all verified in MECs, it would be tangible that airborne PM could alter the biology of MECs (Figure 3). In conclusion, these *in vitro* studies have limitations: 1) PM with different chemical characteristics may elicit different responses in ECs. 2) The most extensively used endothelial cells to study air pollution are macrovascular HUVECs, with limited information on microvascular endothelial cells. 3) MECs have themselves a broad range of phenotypes depending on their tissue of origin, potentially eliciting different responses to environmental stressors (Aird, 2007a; Augustin and Koh, 2017).

### 3.5 Direct *in vivo* and *ex vivo* evidence that wildland fire smoke could impact the microvasculature

As discussed in the above section, *in vitro* studies have shown that PM could impair the structure and function of cultured endothelial cells. Yet, limited evidence exists regarding the direct impact of exposure to wildland fire smoke on the microvasculature *per se* (i.e., arterioles and capillaries). A few pre-clinical studies have investigated the impact of airborne pollution from vehicle exhaust and biomass burning on the mouse microvasculature *in vivo*, suggesting that exposure to wildland fire smoke or air pollution secondary to residential fire overhaul activity post residential fires could induce microvascular alterations (Gainey et al, 2018; Adivi et al, 2021; Scieszka et al, 2021).

Mice exposed to firefighting overhauling activity after a fire that modelled residential fire presented significant changes in the genes expressed within the pulmonary tissue (Gainey et al, 2018). While the authors essentially focused on identifying genes associated with lung carcinogenesis, the KEGG gene ontology analysis also revealed that exposure to the overhaul environment led to a significant over-representation of genes regulated by the Forkhead box O (FoxO) pathway (Gainey et al, 2018). We and others have shown that FoxO signaling pathway is crucial to microvascular homeostasis, where an increased activity of FoxO reduces the capacity of tissues to initiate angiogenesis in response to ischemia or in presence of cardiovascular risk factors (Paik et al, 2007; Milkiewicz et al, 2011; Roudier et al, 2013; Nwadozi et al, 2016; Rudnicki et al, 2018). Further investigations would need to confirm whether exposure to wildland fire smoke could elicit similar effects on the FoxO signaling pathway and impact the capacity of the microvasculature to remodel through angiogenesis.

In another study, Kim and colleagues have exposed mice to PM collected in the vicinity of peat bog fires that took place in the summer 2008 in the rural counties of North Carolina (Kim et al, 2014). Interestingly, the impact of these peat burning events on cardiovascular health was also investigated at the level of the general population by other research groups, and some positive associations between smoke and haze density and the risk of hospital admission for congestive heart failure were identified (Rappold A. G. et al, 2011; Rappold et al, 2012). In their animal study, Kim and colleagues used the Langendorff model to assess the impact of PM exposure on cardiac function. The Langedorff model enables a controlled *ex vivo* perfusion of the heart where ischemia-reperfusion is performed to mimic acute myocardial infarction. It has been previously demonstrated that microvascular alterations were part of the underlying mechanisms leading to ventricular dysfunction and myocardial infarction in the Langendorff model (Hollander et al, 2016). Using this model, Kim and colleagues have shown that female mice exposed to 154 ng/cm^2^ of inhaled ultrafine PM (≤0.1 μm) extracted from peat burning sites had greater infarct size and left ventricular dysfunction post-ischemia when compared to control mice (Kim et al, 2014). These results suggest that inhaling UFP and PM from peat burning smoke could increase the vulnerability of murine hearts to cardiac dysfunction following myocardial infarction, potentially due to greater microvascular injuries (Kim et al, 2014).

The impact of air pollution on the microvasculature has also been investigated at the cerebral level. The brain microvasculature is unique; astroglial cells surround endothelial cells to form a very tight barrier where the exchange of molecules is strictly controlled to protect neurons for toxic substances (Augustin and Koh, 2017). Exposure to vehicle exhaust PM altered the brain’s microvasculature integrity in Apo E^−/−^ mice highly prone to atherosclerosis (Oppenheim et al, 2013; Adivi et al, 2021). The acquisition of a pro-inflammatory profile, an increased microvascular permeability and the associated changes in the expression of tight junction proteins could support the loss of brain microvascular integrity (Oppenheim et al, 2013; Adivi et al, 2021). Exposure to PM from wildland fire origin might also impact the brain microvasculature. Indeed, using a mobile laboratory, Scieszka and colleagues exposed healthy mice to concentrated PM_2.5_ (4 h/day over 20 days) from smoke of wildland fires naturally occurring in California, Arizona, and Washington states (Scieszka et al, 2021). In this study, mice were exposed to an average concentration of 104 μg/m^3^ of PM_2.5_. Animal exposed to these wildland fire related PM_2.5_ had an increased deposition of amyloid β (Aβ-42), a hallmark of cerebrovascular aging, an infiltration of peripheral immune cells, and a shift in the inflammatory profile of the cerebral microvascular endothelium (Scieszka et al, 2021). These changes were also associated to a greater coverage of capillaries by astroglial cells. Thus, PM_2.5_ originating from wildland fire might trigger neuroinflammation and an adverse remodeling of the microvascular blood brain barrier. Further investigations would be required to determine whether these alterations contribute to cerebrovascular aging (Figure 3).

The mentioned studies have investigated the acute or short-term effects of exposure to airborne pollutants from structural fires, biomass burning, and wildland fires. Further studies are however needed to elucidate whether repeated exposure to wildland fire smoke could initiate maladaptive responses of the microvasculature, potentially contributing to or worsening airborne pollution-driven CVD.

## 4 Remaining gaps of knowledge and future directions

Current evidence suggests that inhaled PM_2.5_ and UFP originated from biomass burning or wildland fire smokes could cross the gas-blood barrier to reach microvascular endothelia. Yet, gaps of knowledge remain, and further studies are required to delineate their biological impact on the microvasculature.

First, the chemical composition and size of PM varies depending on the fuel consumed (e.g., biomass vs. gasoline) and the conditions of combustion (Lighty, Veranth, and Sarofim, 2000). To date, most toxicology-based *in vitro* and animal-based *in vivo* studies have investigated the impact of PM from traffic pollution and vehicle exhaust, with only a few studies focusing on PM derived from wildland fire smoke or biomass burning. Most *in vitro* studies also utilized high-to-very high doses of PM to evaluate their toxicity, often far from doses that the general population would be exposed to.

Second, most *in vitro* studies were performed on macrovascular HUVECs, which are phenotypically very different from microvascular MECs (Aird, 2007a; 2007b; Nakato et al, 2019). In mammalians, the vast majority of the endothelia is microvascular (Wagenmakers et al, 2016). The response of MECs to wildland fire-related PM might be considerably different than the responses induced by urban-related PM measured in HUVECs (S. Chen et al, 2017). It appears now crucial to design *in vitro* studies that examine the toxicity of wildland fire-related PM directly on MECs.

Third, very limited data are available regarding the impact of PM exposure from wildland fires on the microvasculature *per se* in animal models. To our knowledge, only two studies have exposed mice to PM naturally collected from forest and peat wildfires (Kim et al, 2014; Scieszka et al, 2021).

Fourth, population-based studies revealed that individuals with conditions or diseases associated to microvascular alterations are more inclined to seek medical assistance due to the worsening of their health status (Johnston et al, 2014; 2007; Rappold A. G. et al, 2011; Rappold et al, 2012; Haikerwal et al, 2015; Yao et al, 2020; Mahsin, Cabaj, and Saini, 2021; Wettstein et al, 2022). Non-invasive assessments of the microvasculature emerge as innovative tools to stratify the cardiovascular risk at a population level. Despite this emergence of these new approaches (Adar et al, 2010; Louwies et al, 2013; Provost et al, 2017; Rijks et al, 2018; Luyten et al, 2020; Korsiak et al, 2021; Witters et al, 2021), the number of studies that investigated the direct impact of outdoor airborne pollution on human microvasculature in the context of wildland fires and biomass burning are insufficient.

In the future, it will be crucial that population-based studies inform the design of preclinical experimental studies to mimic real-world exposure as close as possible. To better determine the vulnerability of microvascular endothelium to wildland fire smoke exposure, experiments must take in consideration the doses of circulating PM, the chemical formulation of PM and their bioavailability in real-world conditions, and the duration of exposure (Lewtas, 2007; Thepnuan et al, 2020). Pre-clinical and clinical studies might help ascertaining whether microvascular alterations are accountable for the adverse cardiovascular outcomes observed in vulnerable populations. Verifying whether wildland fire-related PM directly causes microvascular alterations in these populations requires more intensive work. Undeniably, more population-based studies directly assessing the impact of wildland fire smoke on the microvasculature are needed. Yet, due to the phenotypic differences that exist within the microvasculature, it appears crucial to confirm whether non-invasive assessments of the cutaneous and retinal microvascular beds could correlate with other microvascular impairment observed in other tissues or organs after exposure to PM originated from wildland fire smoke. Careful design of pre-clinical animal studies where the impact of wildland fire PM is tested in multiple microvascular beds (e.g., brain, skeletal muscle, cardiac tissue, retina, and skin) might help in bridging these current gaps of knowledge.

## 5 Conclusive remarks

In a retrospective analysis of the wildland fire seasons 2013, 2015 and 2017–2018 from published Canadian studies (Crouse Dan L. et al, 2012; Stieb et al, 2020), Matz and colleagues estimated the impact on Canadians’ health due to short-term and long term-exposure to wildfire-PM_2.5_ (Matz et al, 2020). They concluded that, each year, there were 50–240 premature deaths due to acute health impact of wildland fire related PM_2.5_, and 570 to 2,500 premature deaths due to chronic health impact. This represents a significant burden on the Canadian health system estimated to cost $410M-$1.8B for the acute impact and $4.3B-$19B for chronic health impact. Most of these health-related costs were attributable to respiratory and cardiovascular conditions (Matz et al, 2020). With the anticipated expansion in size, severity, and number of wildland fires and the predicted lengthening of the fire seasons globally (Bowman et al, 2020; Touma et al, 2021; Senande-Rivera, Insua-Costa, and Miguez-Macho, 2022), better tools are required for cardiovascular health risk assessment. Exploring the biology of the microvasculature in this context could open new avenues for earlier and better detection of adverse cardiovascular outcomes posed by exposure to wildland fire smoke. This will improve knowledge regarding underlying mechanisms of wildland fires’ role in health and will pave the way for better risk management and prevention.

## Figures and Tables

**Table:** 

**Aβ-42**	Amyloid β peptide 42
**Apo E-/- mice**	Atherosclerosis-prone apolipoprotein E-deficient mice
**CHF**	Congestive heart failure
**C57B6J**	mice
**C57**	Black 6 Jackson laboratory mice
**CO2**	Carbon dioxide
**CO**	Carbon monoxide
**DALYs**	Disability-adjusted life years
**EC**	Endothelial cells
**ED**	Emergency Department
**F344 rats**	Fisher 344 rats
**FoxO**	Forkhead box O protein
**GO**	Gene ontology
**H2O**	Water
**HBMEC**	Human brain microvascular endothelial cell
**HMEC**	Human microvascular endothelial cell
**HUVEC**	Human umbilical vein endothelial cell
**ICAM-1**	Intercellular adhesion molecule-1
**ICD9**	International classification of diseases version 9
**IL-1**	Interleukin-1
**KEGG**	Kyoto Encyclopedia of Genes and Genomes
**MEC**	Microvascular endothelial cell
**NOx**	Nitrogen oxide
**NF-κB**	Nuclear Factor Kappa-light-chain-enhancer of activated B cells
**O3**	Ozone
**Ox**	Oxidant capacity of combined O3 and NOx
**ox-LDL**	Oxidized low density protein
**PAH**	Polycyclic aromatic hydrocarbon
**PM**	Particulate matter
**PTSD**	Post-traumatic stress disorders
**RHI**	Reactive hyperemia index
**sICAM**	soluble ICAM
**sVCAM**	soluble vascular cell adhesion molecule 1
**TP53**	Tumor suppressor protein 53
**UFP**	Ultrafine particulate matter
